# Superconducting nanowire single-photon sensing of cerebral blood flow

**DOI:** 10.1117/1.NPh.8.3.035006

**Published:** 2021-08-19

**Authors:** Nisan Ozana, Alexander I. Zavriyev, Dibbyan Mazumder, Mitchell Robinson, Kutlu Kaya, Megan Blackwell, Stefan A. Carp, Maria Angela Franceschini

**Affiliations:** a Massachusetts General Hospital, Harvard Medical School, Optics at Athinoula A. Martinos Center for Biomedical Imaging, Department of Radiology, Boston, Massachusetts, United States; b Massachusetts Institute of Technology, Health Sciences and Technology Program, Cambridge, Massachusetts, United States; c Massachusetts Institute of Technology Lincoln Laboratory, Lexington, Massachusetts, United States

**Keywords:** superconducting nanowire detectors, diffuse correlation spectroscopy, cerebral blood flow, pulsatile blood flow

## Abstract

**Significance:** The ability of diffuse correlation spectroscopy (DCS) to measure cerebral blood flow (CBF) in humans is hindered by the low signal-to-noise ratio (SNR) of the method. This limits the high acquisition rates needed to resolve dynamic flow changes and to optimally filter out large pulsatile oscillations and prevents the use of large source-detector separations (≥3  cm), which are needed to achieve adequate brain sensitivity in most adult subjects.

**Aim:** To substantially improve SNR, we have built a DCS device that operates at 1064 nm and uses superconducting nanowire single-photon detectors (SNSPD).

**Approach:** We compared the performances of the SNSPD-DCS in humans with respect to a typical DCS system operating at 850 nm and using silicon single-photon avalanche diode detectors.

**Results:** At a 25-mm separation, we detected 13±6 times more photons and achieved an SNR gain of 16±8 on the forehead of 11 subjects using the SNSPD-DCS as compared to typical DCS. At this separation, the SNSPD-DCS is able to detect a clean pulsatile flow signal at 20 Hz in all subjects. With the SNSPD-DCS, we also performed measurements at 35 mm, showing a lower scalp sensitivity of 31±6% with respect to the 48±8% scalp sensitivity at 25 mm for both the 850 and 1064 nm systems. Furthermore, we demonstrated blood flow responses to breath holding and hyperventilation tasks.

**Conclusions:** While current commercial SNSPDs are expensive, bulky, and loud, they may allow for more robust measures of non-invasive cerebral perfusion in an intensive care setting.

## 1 Introduction

Diffuse correlation spectroscopy (DCS) is a non-invasive optical method for the measurement of blood flow (BF).^1^ In DCS, the tissue is illuminated by a long coherence length near-infrared laser, and the speckle pattern formed by moving scatterers, mostly red blood cells, modulates the detected light. The decay of the measured temporal intensity autocorrelation function $\left[g_{2}(\tau )\right]$ originated by the speckle fluctuations provides an index of blood flow (${\mathrm{BF}}_{i}$),^2^ with units ${\mathrm{cm}}^{2}/s$. To maximize the contrast of the measured speckle, single-mode fibers are used, greatly limiting potential photon throughput. Current DCS devices employing single-photon avalanche photodiodes (SPAD) detectors and laser sources at 700 to 850 nm typically operate at a source-detector (SD) separation of 25 mm and an acquisition rate of 1 Hz.^3^ Larger SD separations are desirable for improving brain sensitivity and reducing scalp signal contamination, especially in the adult population. Faster acquisition rates are needed to detect fast BF dynamics and effectively remove the large pulsatile systemic component from the cerebral signals. Unfortunately, the low signal-to-noise ratio (SNR) of current devices limits the acquisition rates and prevents the use of SD separations >2.5  cm.

To improve upon the single speckle limitations of conventional DCS measurements, multiple colocalized detectors are used to increase SNR^4^^,^^5^ and as a proof of principle, at a short separation (11 mm), Sie et al.^6^ have recently reported a $g_{2}(4\;\mu s)$ SNR gain of 32 ($\sqrt{1024}$) using a 32×32  pixel SPAD camera to enable multi-speckle detection. As an alternative, DCS measurements with heterodyne detection have been proposed to improve the SNR through amplification of the signal via a reference arm. Using a fiber Mach–Zehnder interferometer and conventional silicon SPAD detectors, we have shown an increase in the SNR of the autocorrelation curve by a factor of ∼2 and a reduction of 80% in the coefficient of variation of the fitted ${\mathrm{BF}}_{i}$ at long source-detector separations (>30  mm).^7^ Further, by increasing the magnitude of the intensity fluctuations, conventional camera sensors can be used, enabling a greater increase in SNR mediated by multi-speckle detection.^8^^,^^9^

In addition to acting at the detection side, we have recently proposed to use wavelengths above 1  μm to increase DCS SNR.^10^ Larger photon availability and slower autocorrelation function decay contribute to a substantial increase in SNR when using wavelengths around 1050 to 1100 nm with respect to the wavelengths traditionally used for near-infrared spectroscopy. The problem of operating at these longer wavelengths is that silicon (Si) SPADs and Si cameras have very low photon efficiencies, indium gallium arsenide (InGaAs) SPADs have a strong afterpulsing probability right where the $g_{2}$ starts to decay (1 to 10  μs), and InGaAs cameras are not fast enough to detect the initial autocorrelation decay.

Here, we propose to use superconducting nanowire single-photon detectors (SNSPDs) to operate DCS at 1064 nm and overcome other detectors limitations.

SNSPDs were demonstrated 20 years ago,^11^ following an observation that the superconductivity of a lead film can be disrupted by a laser beam.^12^ SNSPDs operate below the boiling point of liquid helium, <4.2  K, and consist of a thin film of superconducting material patterned in a compact geometry to create a large pixel with high detection efficiency and a high single-photon sensitivity due to the nanoscale cross-section. When a photon reaches the nanowire, superconductivity is locally broken and the impedance is increased, creating a voltage pulse. After the photon is absorbed, superconductivity quickly recovers and the SNSPD is ready to detect the next photon.

SNSPDs have several advantages over SPADs, such as recovery time (<50  ns), timing precision (<80  ps), photon efficiency (>80%), and broad wavelength sensitivity (600 to 1550 nm).^13^ SNSPDs are also superior to InGaAs SPADs with respect to dark count rate (∼1  CPS versus <10  KCPS) ^14^ and, more importantly, do not have afterpulsing issues. In the following Table 1, we report the key metrics of the detectors used here [single-photon avalanche diode detectors (Si-SPAD) SPCM-NIR-14-FC, Excelitas and SNSPD Opus One, Quantum Opus] and the one of a commercially available InGaAs SPAD (PDM-IR, micro photon detectors).

**Table 1:** Typical key specifications of three single-photon counting detectors used for DCS.

	Dead time	Dark count rate	Timing resolution (ps)	PDE 850 nm (%)	PDE 1064 nm (%)	Operating temperature
**Si-SPAD (excelitas)**	**<25 ns**	**< 1500 CPS**	**350**	**up to 58**	**<3**	**278 to 343 K**
InGaAs SPAD (MPD)	>10 μs^a^	< 10,000 CPS	<130	<5	∼32	228 to 243 K
**SNSPD (quantum Opus)**	**<50 ns**	**1 CPS**	**<80**	**>80**	**>80**	**2 to 3.1 K**

a Required to achieve acceptable afterpulsing probability.

SNSPDs are used in optical quantum information, telecommunication, and space communication.^15^ SNSPDs have also recently been used in fluorescence lifetime imaging microscopy^16^ but never before in biomedical applications. To our knowledge, this is the first application of SNSPDs in humans to improve DCS performance.

In this work, we propose a new DCS system that includes a laser emitting at 1064 nm and two SNSPD detectors. To demonstrate the advantages of the SNSPD-DCS system at 1064 nm with respect to standard DCS, we performed simultaneous measurements on the forehead of 11 subjects with the SNSPD-DCS and an Si-SPAD-based DCS system operating at 850 nm.

## 2 Materials and Methods

### 2.1 Subject Recruitment

For this study, we enrolled 11 healthy subjects (five males, six females, mean age 29±9 years, all right-handed, and non-smokers) between August 2020 and September 2020. The study was reviewed and approved by the Mass General Brigham Human Research Committee (IRB #2019P003074).

### 2.2 SNSPD-DCS System at 1064 nm

**Fig. 1:**
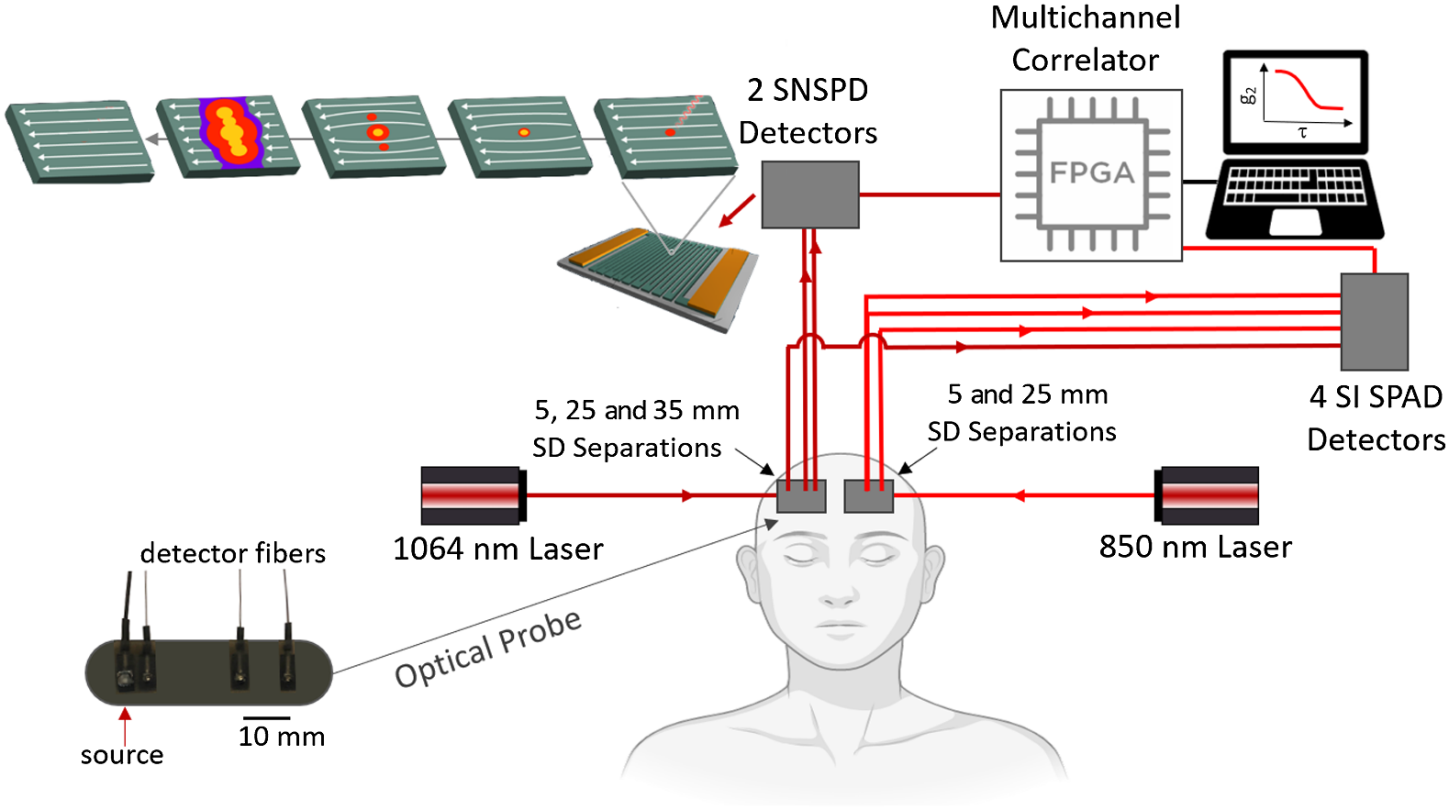
Schematic diagram of the experimental setup. Two long coherence length lasers, one at 1064 nm and one at 850 nm, were used to simultaneously illuminate two locations on the forehead of a subject via multimode optical fibers hosted in two symmetrical optical probes (one shown in details in the low left insert). The probes also included single-mode detector fibers at 5, 25, and 35 mm from the source. For the 850-nm probe we did not use the 35-mm fiber but used two colocalized fibers at 25 mm connected to 2 Si-SPAD detectors to increase SNR. The 5-mm fiber in the 1064-nm probe was connected to a Si-SPAD detector, whereas the 25 and 35 mm separation fibers were connected to two SNSPD detectors. The signals from all detectors was sent to a custom-made FPGA correlator board to digitize the photons arrival times and to a computer to process in real time the temporal autocorrelation function of each detectors and save the data. The figure also shows a graphical rendering of the laser light’s interaction with a SNSPD detector.

The SNSPD-DCS system consists of a long coherence length laser at 1064 nm (CL1064-300-SO, CrystaLaser). At this wavelength, we can illuminate the skin with 100 mW of power. In fact, following the American National Standards Institute for Safe Use of Lasers ANSI Z136.1 2007 Tables 7, 8a, and 8b, pages 77–79, the maximum permitted radiant exposure for continuous skin illumination at 1064 nm is $1\;W/{\mathrm{cm}}^{2}$, and for an illumination spot larger than a 1-mm diameter, a 3.5-mm spot size can be considered, which leads to 100 mW of light power. This power is more than double the allowable power at 850 nm (38 mW when estimated for the same illumination geometry). In the study, light was delivered to the tissue via a 200-μm multimode fiber terminated with a 3.5-mm prism to direct the light perpendicular to the fiber. A holographic diffuser located between the fiber and the prism expanded the beam spot at the skin to about 3-mm OD diameter. The backscattered photons were collected at 5, 25, and 35 mm separations from the source by single-mode fibers (5-μm core diameter, 780HP, Thorlabs) terminated with 1-mm prisms (Fig. 1). The 25- and 35-mm separation fibers were connected to two SNSPDs (Quantum Opus, Opus One) operating at 3.1°K and with direct current of 7  μA. These SNSPDs include a cryostat, which needs to be on for about 3 h to reach the operating low temperature. After that point, the cryostat can be turned off for up to 20 min at a time whenever the device needs to be moved without the need to restart the cooling procedure. The two SNSPD detectors were optimized for 1064-nm illumination by the manufacturer by tuning the detector’s cavity at this wavelength, which led to a photon efficiency of about 88%. For the 5-mm SD separation, we used an Si-SPAD that was part of the conventional DCS system described below since at that distance we had more than enough photons to overcome the very low photon efficiency (<3%) of the Si-SPAD at 1064 nm. The arrival times of the photons were collected by a custom FPGA-based multichannel time-tagger, transmitted to a laptop for real-time display at 1 Hz, and later processed for faster acquisition rates.

### 2.3 Conventional Si-SPAD Based DCS at 850 nm

A conventional DCS system operating at 850 nm was used to compare performances with the SNSPD-DCS system. This custom DCS device included a long coherence length laser (DL852-100-SO, CrystaLaser) attenuated to provide 38 mW at the skin, and four Si-SPAD detectors (SPCM-NIR-14, Excelitas) providing ∼58% photon detection efficiency at 850 nm. An optical probe symmetrical to the one used for the SNSPD-DCS hosted a multimode fiber for the source and single-mode fibers for the detectors, one at 5 mm and two at 25 mm from the source (Fig. 1). The fourth SPAD was connected to the short separation fiber of the 1064 probe.

### 2.4 Measurement Protocols

After a phone screening, on the day of the measurement the subject signed the consent form and was further instructed about the experimental procedures. The subject sat in a testing room adjacent to the control room that housed experimental devices. Both optical probes were channeled through an opening between the rooms and secured to the subject’s forehead as close as possible to the hairline. A pulse oximeter was used to monitor peripheral oxygen saturation (${\mathrm{SpO}}_{2}$) and heart rate (HR). The pulse oximeter and the DCS data were synchronized via computer time-stamps. During the tasks, room lights were dimmed, and due to COVID-19 precautions, the study staff was only in the same room as the subject during the tourniquet task. Subjects wore a surgical mask during all procedures. A glass window between the two rooms allowed us to observe the subject and communication was done via an intercom. The entire session lasted <1  h.

Each subject had one probe on each side of the forehead for the experiments. To account for potential regional differences between the left and right hemispheres, we alternated which side each optical probe was on between subjects. We secured the probes with black tape, which also served to minimize ambient light.

#### 2.4.1 Pressure modulation task

To assess the brain sensitivity of the 1064-nm SNSPD-DCS system, we conducted a pressure modulation task using a medical-grade tourniquet.^17^ The tourniquet was positioned between the eyebrow and the sensors and loosely wrapped around the head. The tourniquet was tightened for 60 s to decrease the blood flow to the scalp by compressing the superficial temporal, the supratrochlear, and the supraorbital arteries. The pressure did not to cause any discomfort to the subjects, and compression was repeated three times, with each trial being preceded and followed by a 1-min baseline.

#### 2.4.2 Breathing task

In this set of measurements, subjects engage in two breathing exercises, breath-holding and hyperventilation. The task consisted of a 1-min baseline, breath-holding for as long the subject was able to but no more than 1 min, a 2-min recovery, 1-min hyperventilation, and a 2-min recovery. For breath-holding, subjects were instructed to begin breathing out 5 s leading up to the task since breath-holding after expiration leads to a more rapid increase in CBF, allowing for shorter breath-holding observation periods.^18^ For hyperventilation, subjects were instructed to attempt to fully exhale and inhale rapidly to maximize the respiratory exchange ratio. The task was repeated three times. Pulse oximeter changes in ${\mathrm{SpO}}_{2}$ and HR were used to assess subject’s compliance.

### 2.5 Data Analysis

For each subject and each trial, at each distance and each wavelength, we computed the temporal autocorrelation functions $\left[g_{2}(\tau )\right]$ at 1 Hz to fit for slow blood flow changes, and at 10, 20, and 50 Hz to extract pulsatile blood flow and to estimate its contrast-to noise ratio (CNR). To calculate ${\mathrm{BF}}_{i}$, each $g_{2}$ was fitted to the semi-infinite correlation diffusion equation^19^ using fixed optical properties corresponding to the ones of the brain layer, reported in Table 2.^20^ The pulsatile CNR was estimated as the contrast between the FFT amplitude at the pulsation frequency and the noise floor, and a clear signal was defined using a threshold of CNR>4.

**Table 2:** Reduced scattering coefficients, absorption coefficients and ${\mathrm{BF}}_{i}$ in four tissue layers derived from Refs. 20, 30, and 31 and used in the Monte Carlo simulations. For the SNR evaluation, we used the brain optical properties.

Optical properties tissue layer	${\mu_{s}}^{\prime }$ (${\mathrm{cm}}^{-1}$)		$\mu_{a}$ (${\mathrm{cm}}^{-1}$)		
	1064 nm	850 nm	1064 nm	850 nm	${\mathrm{BF}}_{i}$ (${\mathrm{cm}}^{2}/s$)
Scalp	8.38	9.25	0.11	0.091	$1\times 10^{-8}$
**Skull**	**8.38**	**9.25**	**0.13**	**0.11**	**0**
CSF	0.09	0.10	0.12	0.043	0
**Brain**	**8.38**	**9.25**	**0.17**	**0.20**	$6\times 10^{-8}$

For the pressure modulation and breathing task comparisons, we considered relative blood flow (${\mathrm{rBF}}_{i}$), obtained in each subject and in each trial by normalizing ${\mathrm{BF}}_{i}$ by the mean value calculated between 10 and 50 s of the baseline period.

We calculated ${\mathrm{BF}}_{i}\%$ reductions with pressure as $(1-{\mathrm{rBF}}_{i\;\text{pressure}})*100$. Since the two DCS optical sensors were not colocalized, we considered only trials for which the difference between the left and right forehead’s reductions at the short separations was small (<10% difference) for a period of at least 20 s during the compression.

To assess subject compliance with the breathing tasks, we examined the pulse oximetry responses. For the breath-holding task, we divided the trials in two groups based on ${\mathrm{SpO}}_{2}$ decreases lower or >5%. For the hyperventilation trials, we excluded subjects where we did not observe an increase in hearth rate of at least 10 beats per minute. Each trial was normalized with respect to a 30 s baseline before breath-holding and hyperventilation.

In addition to ${\mathrm{BF}}_{i}$, average photon counts at both wavelengths and all separations were quantified. Gains at the same SD separations were defined as the ratio between the value at 1064 and the value at 850 nm.

To verify the tabulated difference in scattering between 850 and 1064 nm matched the experimental results we assumed the same blood flow and a fixed absorption coefficient ($\mu_{a}$) at each wavelength. Specifically, we first imposed a constant reduced scattering coefficient (${\mu_{s}}^{\prime }$) of $9.25\;{\mathrm{cm}}^{-1}$ to fit the 850 nm autocorrelation curve and extracted the 850 nm ${\mathrm{BF}}_{i}$. Then, we fit the $g_{2}$ curve at 1064 nm with scattering values ranging from 6.5 to $10.5\;{\mathrm{cm}}^{-1}$ and found which ${\mu_{s}}^{\prime }$ provided the minimum difference between ${\mathrm{BF}}_{i}$ at 1064 nm and ${\mathrm{BF}}_{i}$ found at 850 nm.

To evaluate the SNR of the two DCS devices in the most realistic situation, we decided to use human data. For this SNR evaluation, we used the three 1-min baseline periods acquired at the beginning of the pressure modulation tasks. To eliminate physiological noise, we only considered $g_{2}$ values at the diastolic points of the cardiac cycle. The diastolic points were identified from the 10 Hz ${\mathrm{BF}}_{i}$ time trace at both 850- and 1064-nm short separations, and at 25 mm at 1064 nm since in these cases the pulsatile CNR was >4. The identified diastolic timepoints were used in all ${\mathrm{BF}}_{i}$ traces, including the 25 mm at 850 nm and of 35 mm at 1064 nm where the higher noise made the diastolic points unidentifiable. The $\text{mean}[g_{2}(\tau )]$ and the standard deviation of $\left[g_{2}(\tau )\right]$ were calculated at a correlation lag time τ=4  μs by averaging 50 dyastolic $g_{2}$ acquired at 10 Hz. Finally, the SNR was calculated using the following expression:

$$\mathrm{SNR}|g_{2}(\tau )|=\text{mean}[g_{2}(\tau )-1]/\mathrm{STD}[g_{2}(\tau )].$$

We also compared the experimental SNR with the theoretical SNR as described in the noise model in Refs. 21 and 22. Based on this model, the theoretical standard deviation [σ(τ)] at each time delay τ is given by

$$\sigma (\tau )=\sqrt{\frac{T}{t}}[\beta^{2}\frac{(1+e^{-2\Gamma T})(1+e^{-2\Gamma \tau })+2m(1-e^{-2\Gamma T})e^{-2\Gamma \tau }}{\left(1-e^{-2\Gamma T}\right)}+2{\left\langle n\right\rangle }^{-1}\beta (1+e^{-2\Gamma \tau })+{\left\langle n\right\rangle }^{-2}(1+\beta e^{-\Gamma \tau }){]}^{1/2},$$

where t is the integration time; T is the bin width; Γ is the exponential decay rate of $g_{1}(\tau )$, which depends on ${\mathrm{BF}}_{i}$; ⟨n⟩ is the average number of recorded photons multiplied by the bin width T; m is the is auto-correlation lag bin index; and β is the coherence factor. Actual photon counts and average β and ${\mathrm{BF}}_{i}$ were inputs to the model, and the same brain optical properties used for the experimental calculations were used for the noise model.

Moreover, we evaluated the expected brain sensitivity with Monte Carlo simulations. The MCX software package^23^ was used to simulate photon transport and momentum transfer in a realistic brain geometry.^24^ For this forward model, we used a four-layer MRI-derived volumetric geometry segmented into scalp, skull, cerebrospinal fluid (CSF), gray, and white matter (brain), with 1  mm×1  mm×1  mm spatial resolution. For each of the four tissue types, we used the optical properties and ${\mathrm{BF}}_{i}$ reported in Table 2. The probe consisted of a collinear arrangement of a source and seven detectors at distances (r)=5, 10, 15, 20, 25, 30, 35, and 40 mm from the source and was placed in a location in the head with extracerebral thickness of about 14 mm. Auto-correlation curves obtained from MC simulations were post-processed to add statistical noise using the same noise model mentioned in the previous section,^21^ then fit with the same semi-infinite correlation diffusion model as the experimental data.

For the photon counts, we used the experimental values averaged across all subjects: 114,000 counts per second (CPS) at 25 mm for 1064 nm, 11,000 CPS at 25 mm 850 nm, and 12,000 CPS at 35 mm at 1064 nm. To estimate brain sensitivity, we increased the ${\mathrm{BF}}_{i}$ in the brain layer by 20% to $7.2\times 10^{-9}\;{\mathrm{cm}}^{2}/s$ (${\mathrm{BF}}_{i\;\text{perturb}}$) while keeping scalp, skull and CSF ${\mathrm{BF}}_{i}$ constant and quantified the increase in apparent ${\mathrm{BF}}_{i}$ fit with a semi-infinite model. To estimate ${\mathrm{BF}}_{i}$ reduction due to scalp contamination at different SD separations, we simulated a scalp BF reduction of 85% (as found in our pressure modulation experiments) and quantified the decrease in apparent ${\mathrm{BF}}_{i}$ fit with the same semi-infinite model. To determine the brain contrast-to-noise ratio at each SD separation (r) and wavelength (λ), we used the perturbation and baseline estimated ${\mathrm{BF}}_{i}$ from fitting and estimated the standard deviation of fitted ${\mathrm{BF}}_{i}$ over 120 noise realizations assuming a 1-s integration time. The brain CNR was estimated for each experimental SD separation and wavelength as

$$\mathrm{CNR}(r,\lambda )=\frac{BF_{i\;\text{perturb}}(r,\lambda )-BF_{i\;\text{baseline}}(r,\lambda )}{\sigma_{BFi}(r,\lambda )}.$$

## 3 Results

### 3.1 SNR Comparison

**Fig. 2:**
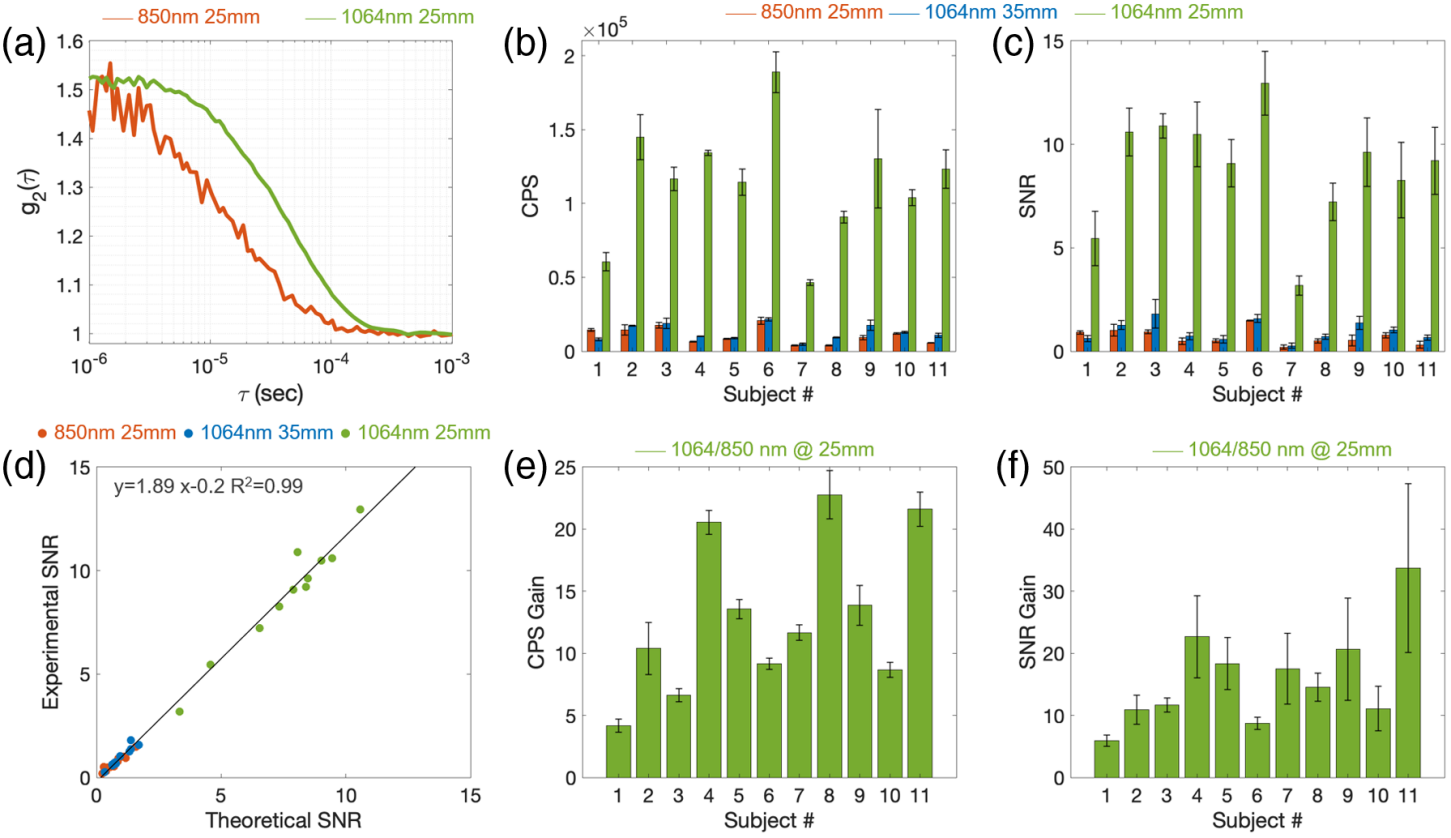
(a) Autocorrelation curves acquired at 50 Hz and averaged over 50 diastolic points at 850 and 1064 nm, both at a 25-mm SD separation, in a representative subject (#6). The $g_{2}$ at 1064 nm is much less noisy and shows a slower decay, which further improves the SNR. (b) and (c) CPS and $g_{2}(4\;\mu s)$ SNR in the 11 subjects at the three large separations. (d) Experimental versus theoretical $g_{2}(4\;\mu s)$ SNR for all subjects. (e) and (f) CPS gain and $g_{2}(4\;\mu s)$ SNR gain at 1064 nm 25 mm with respect to 850 nm at 25 mm. In all bar-graphs each value represents the average and each errorbar represent the standard deviations across the three trials. In all panels, orange correspond to 850 nm at 25 mm, blue correspond to 1064 nm at 35 mm, and green correspond to 1064 nm at 25 mm.

As described in Sec. 2.5, to assess the SNR of the SNSPD-DCS with respect to conventional DCS, we considered the baseline periods of the pressure modulation trials and averaged 50 $g_{2}$ curves acquired at 10 Hz at the diastolic point of the arterial pulsation cycle. Figure 2(a) shows the resulting autocorrelation curves at 25 mm for the 1064 nm and the 850 nm DCS systems in a representative subject. As expected, the autocorrelation curve at 1064 nm has a slower decay than the $g_{2}$ at 850 nm due to the lower scattering and longer wavelength. By fixing ${\mu_{s}}^{\prime }$ at 850 nm to $9.25\;{\mathrm{cm}}^{-1}$, we obtained an average ${\mathrm{BF}}_{i}$ of $1.3\pm 0.5\times 10^{-8}\;{\mathrm{cm}}^{2}/s$ and a reduced scattering coefficient at 1064 nm of $8.39\pm 1.40\;{\mathrm{cm}}^{-1}$, very close to the one reported in Table 2. Since the measurements were not colocalized in each subject, some differences between the two wavelengths are expected. Figures 2(b) and 2(c) report the average CPS and $g_{2}(4\;\mu s)$ SNR in each subject at the large SD separations for the two DCS systems. Figure 2(d) reports the theoretical and experimental $g_{2}(4\;\mu s)$ SNRs, showing high correlations between theoretical and experimental SNR at all wavelengths and SD separations (R2=0.92 for 850 nm at 25 mm, R2=0.9 for 1064 nm at 35 mm, and R2=0.95 for 1064 nm at 25 mm). Finally, Figs. 2(e) and 2(f) show the CPS and $g_{2}(4\;\mu s)$ SNR gain of 1064 nm with respect to 850 nm at a 25 mm SD separation. At the same separation, SNSPD-DCS collected an order of magnitude more photons than conventional DCS (average gain 13±6) and for τ=4  μs we achieved an SNR gain of 16±8. For τ ranging between 1 and 10  μs the average SNR gain was 18±10. Moreover, with SNSPD-DCS, we were able to perform measurements at 35-mm separation while achieving a similar CPS and SNR as conventional DCS at 25 mm. It is worth noting that in female subjects (#2, 3, 6, 9, 10, and 11), we collect an average of 1.8±0.2 times more photons and achieve 1.8±0.4 times higher SNR than in male subjects, with the highest CPS and SNR gain at 1064 at 35 mm.

**Fig. 3:**
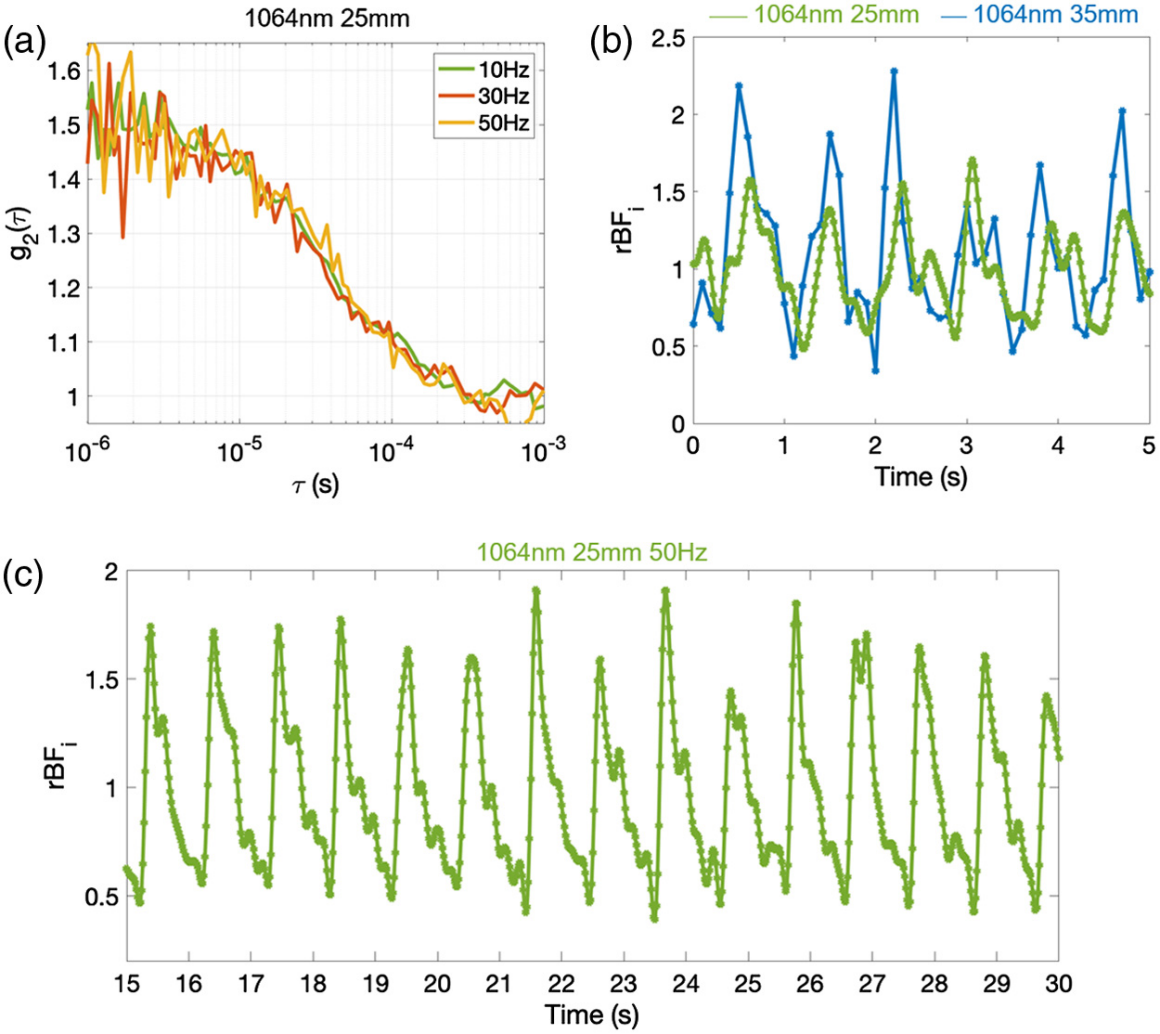
(a) Example of an autocorrelation function acquired with the SNSPD-DCS at 25 mm, estimated at three different frequencies, 10 Hz (100 ms integration time), 30 Hz (33 ms), and 50 Hz (20 ms), on subject #6. While the noise increases with frequency, the decay rate and the resulting ${\mathrm{BF}}_{i}$ fit are consistent at all acquisition rates. (b) For the same subject as panel (a), we show 5 s of the ${\mathrm{BF}}_{i}$ traces sampled at 50 Hz and 25 mm and at 10 Hz and 35 mm SD separations. (c) Example of ${\mathrm{BF}}_{i}$ acquired at 50 Hz and 25 mm in subject # 5 (SNR=9). A Butterworth bandpass filter of 0.1 to 5 Hz was applied to the data.

The increased $g_{2}$ SNR allowed us to acquire data at 25 mm at faster rates and recover clean arterial pulsation signals at 20 Hz in all subjects (FFT pulsatile component CNR>4). At 35 mm and 1064 nm and at 25 mm and 850 nm, we achieved pulsatile CNR>4 in only four subjects (36%) and only at 10 Hz acquisition rate. In these subjects, the CNR at 850 nm was 5.6±1.3, lower than the CNR at 1064 nm (10±3.8) at the same SD separation, 25 mm. Figure 3 shows examples of $g_{2}$ autocorrelation curves acquired at 10, 30, and 50 Hz, and pulse waveforms at 25 and 35 mm with the SNSPD-DCS system.

### 3.2 Sensitivity Estimates via Monte Carlo Simulation

**Fig. 4:**
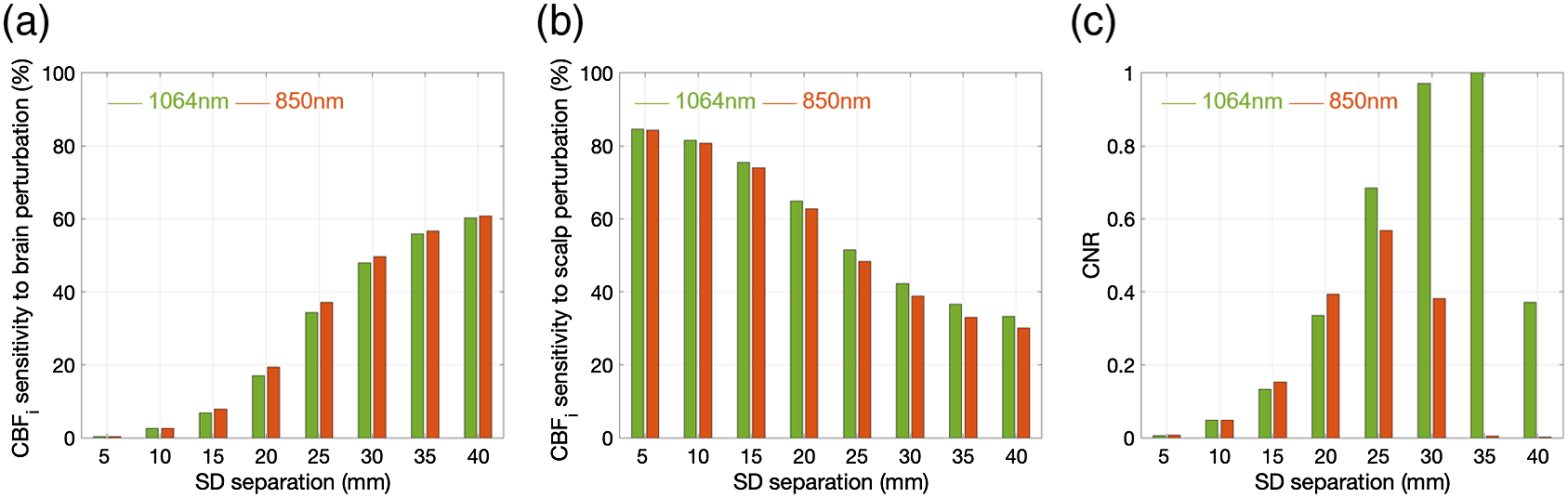
CBF index sensitivity as a function of SD separation at 850 and 1064 nm for (a) a brain perturbation (20% increase in CBFi) and (b) a scalp perturbation (85% increase in scalp BFi). (c) Brain contrast-to-noise ratio (normalized by the maximum value) as a function of SD separation at 850 and 1064 nm at 1 Hz acquisition rate.

The Monte Carlo simulations on a 3D segmented head structure, as expected, showed increased sensitivity to the brain with increasing SD separations. As shown in Figs. 4(a) and 4(b), there is no substantial difference between cerebral blood flow (CBF) sensitivity at 850 and 1064 nm for the chosen optical properties (Table 2) when noise effects are not taken into account. Cerebral ${\mathrm{BF}}_{i}$ sensitivity increases substantially at 35 mm with respect to the 25-mm SD separation with an increase of 21.65% for a brain perturbation (20% increase in ${\mathrm{CBF}}_{i}$) and a decrease of 15% for a scalp ${\mathrm{BF}}_{i}$ perturbation (85% scalp BFi reduction) at 1064nm. This is in agreement with our experimental results where for averaged data (acquisition rate 0.1 Hz) we did not observe significant differences in ${\mathrm{BF}}_{i}$ changes at 25 mm at the two wavelengths, but we observed significant improvements at 35-mm SD separation, only achievable at 1064 nm (SNR at 850 nm is too low at this separation even when acquiring at 0.1 Hz or less). We then add noise to the simulations based on the CPS obtained experimentally at 25- and 35-mm SD separations, and compute the expected contrast-to-noise-ratio of CBF measurement. As shown in Fig. 4(c) (CNR normalized by the maximum value) the improvement in the performance of the SNSPD-DCS at 1064 nm with respect to the Si-SPAD-based DCS at 850 nm are clear. As separation increases, noise rises much faster than intrinsic sensitivity, leading to an optimal separation range for each wavelength. This optimal SD separation at 850 nm is 25 mm, whereas for the SNSPD-DCS at 1064 nm, the optimal SD separation is 35 mm, which allows for a 31.6% relative increase in brain blood flow sensitivity.

### 3.3 Pressure Modulation Results

**Fig. 5:**
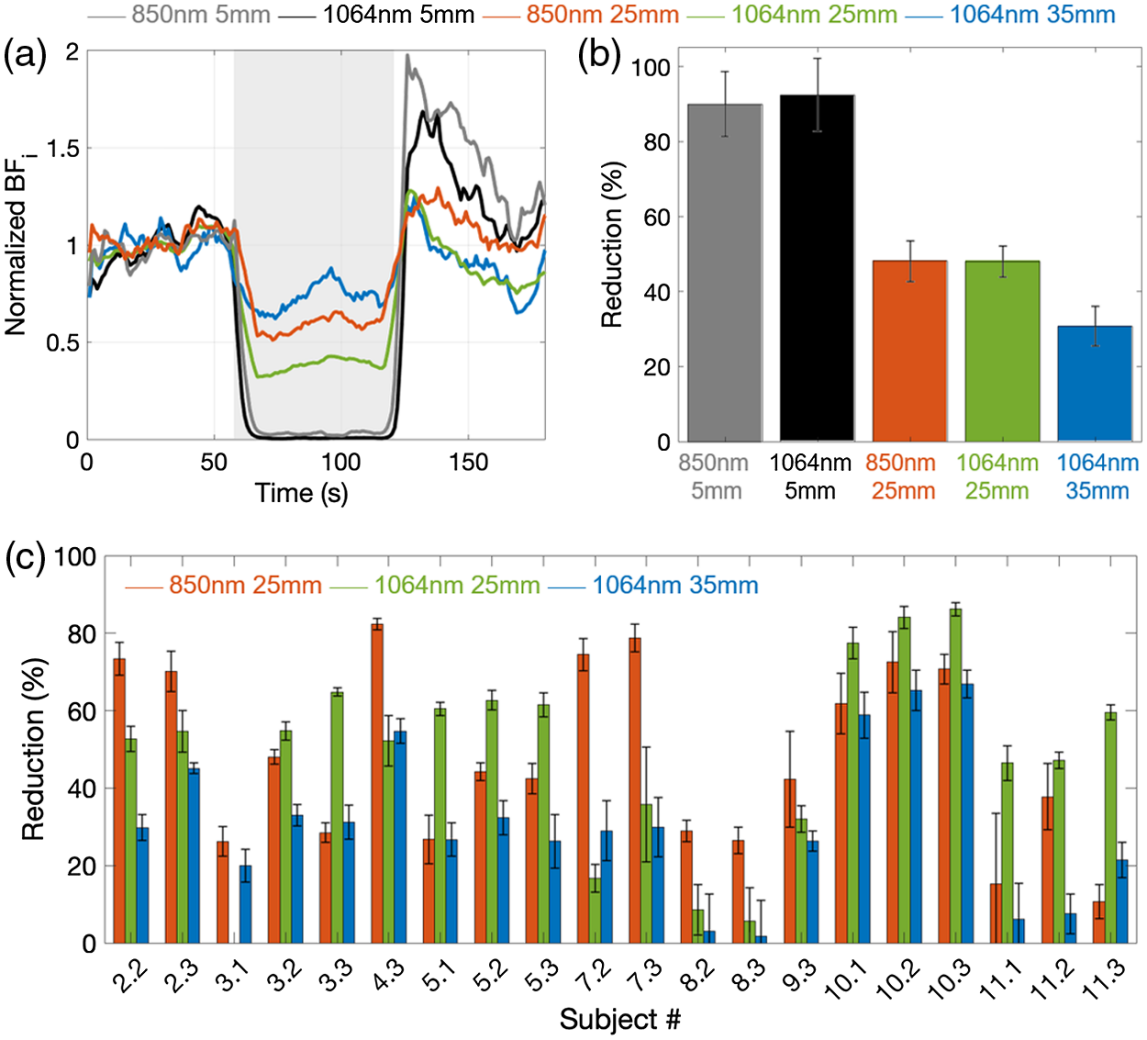
(a) An example of a pressure modulation trial (subject #5, trial #3), reporting ${\mathrm{rBF}}_{i}$ at 1 Hz for all SD separations of the two DCS systems. The gray shaded area represents the period during which the tourniquet was tightened. (b) Percent reduction in ${\mathrm{rBF}}_{i}$ during pressure with respect to initial baseline at all SD separations averaged across all trials and subjects. Error bars represent standard errors across all subjects. (c) Percent reduction in ${\mathrm{rBF}}_{i}$ during pressure with respect to initial baseline at the large SD separations for each included trial. Error bars represent standard deviations during the compression periods.

Subjects #1 and #6 and trials 2.1, 4.1, 4.2, 7.1, 8.1, 9.1, and 9.2 were excluded based on the criteria defined in Sec. 2.5 (a >10% difference in the reduction of the two short separation wavelengths). No statistical difference was found on the remaining trials between the short separations at 1064 and 850 nm, with an average reduction at the 5 mm SD separations of 89.4±4.5 at 850 nm and of 93.4±3.7 at 1064 nm (p=0.08). Figure 5(a) shows an example of ${\mathrm{rBF}}_{i}$ versus time during a trial (subject #5 trial #3). Figure 5(b) and 5(c) report the average reductions for each trial and the grand averages across all trials. While we did not find statistically significant differences between the reductions at 25 mm for either wavelength (48.1±7.7 and 48.0±8.4, p=0.99), we consistently observed a lower reduction for the 35 mm at 1064 nm measurements, equal to 30.7±6.4, with $p=3.6\times 10^{-4}$ for the comparison with 25 mm at 850 nm, and $p=1.52\times 10^{-4}$ for the comparison with 25 mm at 1064 nm. The experimental result is in good agreement with the Monte Carlo simulations [Fig. 4(b)]. At 25-mm SD separation, the experimental ${\mathrm{BF}}_{i}$ reduction of 48% and 48.1%, at 850 and 1064 nm, respectively, match the simulation reductions (48.1% and 51.5%, at 850 and 1064 nm, respectively). At 35 mm and 1064 nm, the experimental reduction of 30.7% is also similar to the simulation ${\mathrm{BF}}_{i}$ reduction (36.5%).

As shown in Fig. 5, the main advantage of SNSPD-DCS is that we are able to perform measurements at larger SD separations and increase the sensitivity to cerebral changes in all subjects.

**Fig. 6:**
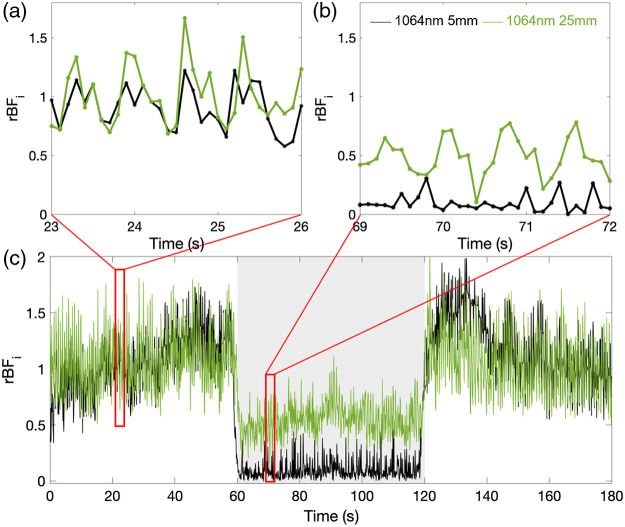
${\mathrm{rBF}}_{i}$ during a pressure modulation trial on a representative subject (#11.1) acquired at 10 Hz. Results at 5- and 25-mm SD separations at 1064 nm are reported in figures. The large fluctuation on the signal is not noise but the pulsatile blood flow oscillations. Panels (a) and (b) give an expanded view of two 3-s periods during baseline and pressure.

In addition to a reduction in ${\mathrm{BF}}_{i}$, the compression also drastically reduced the pulsatile waveform at the short separations. Conversely, the amplitude of the pulsatile blood flow at the larger separations remains substantial, with an average reduction of 35.5±29% at 25 and 1064 nm. An example is reported in Fig. 6. This suggests that a large component of the pulsatile ${\mathrm{BF}}_{i}$ signal originates below the scalp and skull.

### 3.4 Breathing Tasks Results

These experiments were conducted to induce and measure differences in cerebral versus peripheral responses to systemic perturbations. Subjects 4 and 8 (except 8.3 breath-holding) were excluded because of large oscillations of the ${\mathrm{BF}}_{i}$ at short separations. Subject 9 did not perform the first hyperventilation trial. For breath-holding, we divided the trials in two groups. In the first group, we measured an ${\mathrm{SpO}}_{2}$ decrease in response to breath-holding of ≥5% (1.1, 1.2, 1.3, 2.2, 3.1, 5.2, 6.1, 6.2, 6.3, 8.3, 11.1, 11.2, 11.3; 13 trials). The second group had trials for whom ${\mathrm{SpO}}_{2}$ responses <5% (2.1, 2.3, 3.2, 3.3, 5.1, 5.3, 7.1, 7.2, 7.3, 9.1, 9.2, 9.3, 10.1, 10.2, 10.3; 15 trials). For the hyperventilation results, we excluded trials 2.3 and 9.3 because the HR changed <10  bpm as a consequence of the fast breathing, leaving us with 24 trials.

**Fig. 7:**
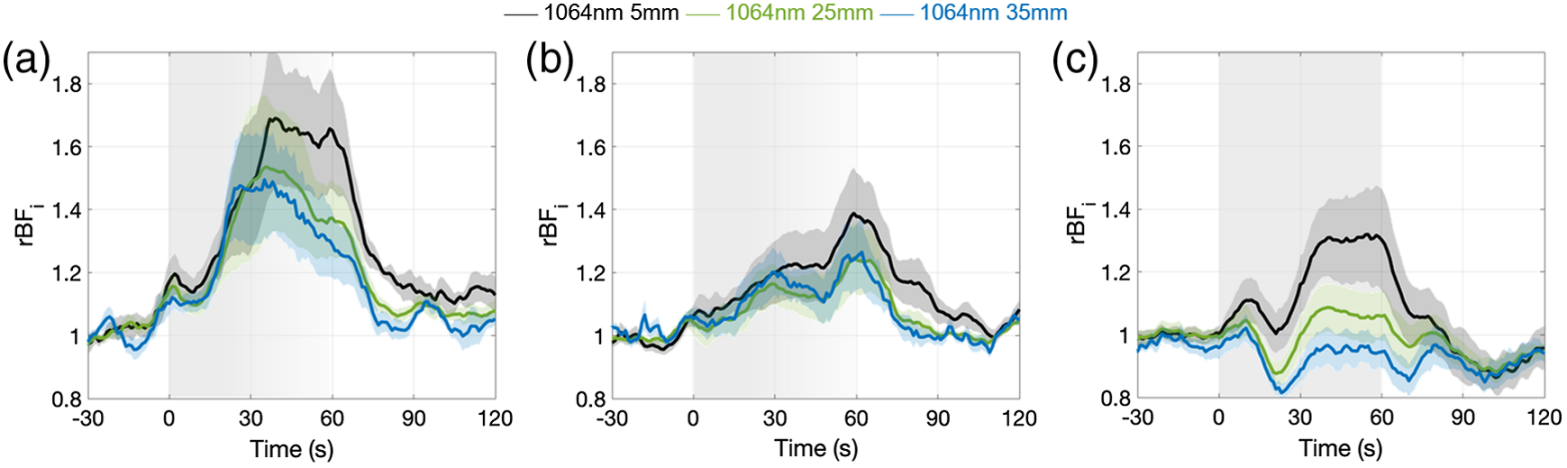
Breathing tasks ${\mathrm{BF}}_{i}$ results. (a) ${\mathrm{BF}}_{i}$ changes during breath-holding task averaged across group 1 trials (${\mathrm{SpO}}_{2}$ reduction ≥5%). (b) ${\mathrm{BF}}_{i}$ changes during breath holding task for group 2 (${\mathrm{SpO}}_{2}$ reduction <5%). (c) ${\mathrm{BF}}_{i}$ changes during hyperventilation average across all included trials. Time zero correspond to the start of breath-holding or hyperventilation. While all subjects performed a breath-holding of at least 25 s, only few were able to maintain it for 60 s (shown as a gray gradient shaded period in the figures). For hyperventilation, all subjects performed it for 60 s. Shaded areas represent standard errors across subject.

Figure 7 reports the relative ${\mathrm{BF}}_{i}$ changes for the different groups and tasks measured with the SNSPD-DCS system at 1064 nm, with Figs. 7(a) and 7(b) reporting the ${\mathrm{rBF}}_{i}$ responses to the breath-holding task in groups 1 (${\mathrm{SpO}}_{2}$ drop≥5%) and 2 (${\mathrm{SpO}}_{2}$ drop <5%) and Fig. 7(c) reporting ${\mathrm{rBF}}_{i}$ changes with hyperventilation. As expected, blood flow increases during the first 30 s in response to breath-holding due to transient hypercapnic hypoxia and slowly returns to baseline during the second phase of breath-holding due to a high concentration of ${\mathrm{CO}}_{2}$ in the blood stream and a deficiency of $O_{2}$ in the tissues. Larger changes were observed in the group with larger ${\mathrm{SpO}}_{2}$ drops (69±19.3% in group 1 versus 38±9.6% in group 2). In both panels a and b, we can see faster responses at larger separations, due to the faster reactivity of the brain than the scalp.^25^ Statistically significant differences between ${\mathrm{rBF}}_{i}$ at 35 and 5 mm SD separations were found at times 63 to 88 s and 108 to 118 s (p<0.05), Statistically significant differences between ${\mathrm{rBF}}_{i}$ at 25 and 5 mm were found at times 63 to 84 s and 108 to 109 s (p<0.05). No statistically significant differences were found between responses at 35 and 25 mm.

The hyperventilation task causes hypocapnia, and while the scalp blood flow increases due to the large increase in HR during fast paced breathing, the CBF decreases due to vasoconstriction. As shown in Fig. 7(c), we clearly observed this differential behavior between scalp and brain in the ${\mathrm{rBF}}_{i}$ responses at 5 and 35 mm, with responses at 25 mm in between the two, because of the lower sensitivity to brain at this SD separation. Specifically, ${\mathrm{rBF}}_{i}$ at 5 mm showed an increase of 31.9±13.8% and rBFi at 35 mm showed a decrease of 18.5±2.3%. Statistically significant differences between ${\mathrm{rBF}}_{i}$ at 35 and 5 mm SD separations were found at times 0 to 78 s (p<0.05). Statistically significant differences between ${\mathrm{rBF}}_{i}$ at 25 and 5 mm between 0 and 72 s. Statically significant differences between ${\mathrm{rBF}}_{i}$ at 25- and 35 mm-SD separations were found at times 24 to 85 s (p<0.05).

Breath-holding and hyperventilation responses at 850 nm had similar trends, with within and across subject’s differences due to the different measurement locations at the two wavelengths (not shown). We quantified the ${\mathrm{rBF}}_{i}$ changes at 850 nm and 1064 nm (at the same SD separation, 25 mm) during the breathing tasks via Bland–Altman analysis. For the maximum ${\mathrm{rBF}}_{i}$ changes in the breath-holding groups I and II and in the hyper-ventilation (24 trials), 94.23% (49 out of 52) of the data points (i.e., relative changes at 1064 and 850 nm) fall within ±1.96 STD of the mean difference, due to the similar trends between the two wavelengths.

## 4 Discussion and Conclusions

This is the first report demonstrating a high signal-to-noise ratio with homodyne DCS at an SD separation of 35 mm *in-vivo* on a substantial number of subjects. As described in Sec. 2, the advantages of the presented method were achieved by using a single channel SNSPD and 1064 nm illumination. The major contribution to the SNR improvement with respect to conventional DCS is given by the 7 to 8 times more photons available at the detector at 1064 than at 850 nm, as reported in Ref. 10. An additional contribution to the total photon gain at 1064 with respect to 850 nm is given by the 1.5 times higher photon detection efficiency (PDE) of the SNSPD used here (∼88% at 1064 nm) and the extremely low dark count rate (1 CPS) with respect to the Si-SPAD PDE (∼58% at 850 nm) and dark count rate (1500 CPS). Together, these two factors provide an overall experimental averaged gain of 13 in photon count at 25-mm SD separation and, by also considering the slower decay of the autocorrelation function at the longer wavelength, an averaged $g_{2}(4\;\mu s)$ SNR gain of 16. It is worth noting that $g_{2}$ SNR increases in direct proportionality to the instantaneous count rate, whereas it only increases with the square root of the acquisition time or multi-channel averaging. Thus, for standard DCS, to achieve the same $g_{2}$ SNR gain achieved here one would need to increase the integration times or the number of channels by a factor of 256 ($∼16=\sqrt{256}$).

By using SNSPD also at 850 nm the photon budget improvement at 1064 nm versus 850 nm would have been 1.5 time smaller than what measured using Si-SPADs at 850 and SNSPD at 1064 nm. Using Si-SPAD at 1064 nm would have drastically reduced the 1064 photon budget since the PDE of the Si-SPAD at 1064 is <3%.

The increased SNR of the SNSPD-DCS at 1064 nm allowed us to resolve clear arterial pulsation at 20 Hz at a 25 mm SD separation in all subjects. Moreover, as previously observed by Wang et al.,^26^ we found the pulsatile blood flow component at this SD separation is less affected by scalp interference than the average blood flow signal as shown during the pressure modulation experiment. In fact, during compression at 25 and 1064 nm we observed a ${\mathrm{BF}}_{i}$ reduction of 48.1% and an attenuation of pulsatile flow of 35%. Being able to acquire pulsatile blood flow at high frequency with low SNR at separations of 25 mm or more, is important in applications where pulsatile blood flow is used to estimate intracranial pressure non-invasively.^17^^,^^27^

In agreement with the Monte Carlo simulations, we observed improved brain sensitivity in our experiments by increasing SD separation. The pressure modulation results show a smaller reduction in ${\mathrm{rBF}}_{i}$ at 35 mm separation with respect to 25 mm (both 850 and 1064 nm) and the breathing tasks shows more consistent statistically significant differences in ${\mathrm{rBF}}_{i}$ between 5 and 35 mm. ${\mathrm{rBF}}_{i}$ increases of 69% during breath-holding and decreases of 18.5% during hyperventilation are in agreement with MRI and PET studies.^25^^,^^28^^,^^29^

While in some subjects we obtain similar responses at 25 and 35 mm, the use of 35 mm allows recovery of CBF changes more consistently across subjects. This is needed to provide better accuracy and consistent efficacy when moving to adult clinical applications.

## Code, Data, and Materials Availability

Data supporting the results reported in the manuscript may be requested by contacting the corresponding author.
